# Bone Marrow Myeloid–Lymphatic Progenitors Expand Tumor Lymphatic Vasculature Through Cell Fusion

**DOI:** 10.3390/cancers17111804

**Published:** 2025-05-28

**Authors:** Shaswati Athaiya, Lisa Volk-Draper, Emma Cox, Kathy Robinson, Natalya Zinkevich, Sophia Ran

**Affiliations:** 1 Department of Medical Microbiology, Immunology, and Cell Biology, Southern Illinois University School of Medicine, Springfield, IL 62794, USA; sathaiya62@siumed.edu (S.A.); lisa.volk-draper@llcc.edu (L.V.-D.); ecox12@utexas.edu (E.C.); 2 Simmons Cancer Institute at SIU School of Medicine, Springfield, IL 62702, USA; krobinson@siumed.edu; 3 College of Health, Science, and Technology, School of Integrated Sciences, Sustainability, and Public Health, University of Springfield, Springfield, IL 62703, USA; nzink2@uis.edu

**Keywords:** breast cancer, lymphangiogenesis, lymphatic metastasis, bone marrow, myeloid–lymphatic progenitors, cell fusion, chimera models, TLR4, Th2 cytokines

## Abstract

**Background:** Bone marrow (BM)-derived myeloid–lymphatic endothelial cell progenitors (M-LECPs) promote formation of tumor lymphatics that are responsible for metastasis to lymph nodes. The regenerative capacity of BM progenitors to other lineages is mediated through cell fusion, a process that delivers a pro-mitotic message directly to division-restricted cells. This suggested that M-LECPs might use a similar mechanism to induce division of lymphatic endothelial cells (LECs). Methods: To test this hypothesis, we determined expression of fusogenic markers in M-LECP produced in vitro and recruited to human or mouse tumors in vivo as well as quantified their fusion with LECs in both settings. Fusion in vivo was determined in female chimera mice grafted with male BM that have been implanted with MDA-MB-231 or EMT6 breast tumors. Co-staining for Y-chromosome and LEC-specific markers allowed us to quantify tumor lymphatic vessels fused with BM progenitors. Results: We found that both tumor-recruited and in-vitro-produced M-LECPs expressed multiple fusogenic regulators and possessed a significant fusogenic activity towards cultured and vessel-lining LECs. Y-chromosomes, a marker of fusion, were detected in nearly half of tumor lymphatics and were associated with mitotic division, vessel formation, and node metastasis. Both in vitro and in vivo assays showed dependency of fusion on Th2 and Toll-like receptor-4 (TLR4) pathways. Conclusions: This novel mechanism of tumor lymphatic formation triggered by fusion with BM myeloid–lymphatic progenitors suggests a variety of new targets for inhibition of metastatic spread.

## Simple Summary

Lymphatic vessels significantly contribute to breast tumor spread. Cells from the bone marrow called lymphatic endothelial progenitors promote generation of these vessels. Progenitors to other cell types are known to promote tissue revival by transferring their contents to damaged cells which leads to cell division. We hypothesized that pro-lymphatic progenitors use the same mechanism of fusion to induce new lymphatic vessels. We found that lymphatic progenitors express proteins that allow merging with vascular endothelial cells and that this event is regulated by immune factors known to promote tumor spread. These findings offer new understanding of creation of new tumor vessels that are directly responsible for cancer spread. This can aid development of new treatments to prevent tumor spread, leading to improved survival of cancer patients.

## 1. Introduction

The lymphatic system plays a major role in normal physiology and tumor pathology. Under normal conditions, lymphatic vessels regulate fluid and protein interstitial balance [1], transport leukocytes to and from lymph nodes (LNs) [2], and promote tissue repair through debris clearance [3] and removal of immunostimulatory cells [4]. In the context of cancer, tumor-induced lymphatic vessels contribute to metastasis by transporting tumor cells to regional LNs. The prerequisite for LN metastasis is tumor-induced lymphangiogenesis, i.e., the formation of new lymphatic vessels [5]. Metastatic cells in LNs are the source for distant metastasis [6,7], which is the main cause of mortality from cancer [5]. Consequently, tumor lymphatic vessel density (LVD) and positive LN status are strong predictors of poor survival of patients with breast cancer (BC) [5] and other tumors [8]. It is therefore of prime clinical interest to delineate the mechanisms of tumor lymphatic formation which enhances LN metastasis, and ultimately, lethal distant spread.

The main mechanism inducing lymphangiogenesis is currently thought to be mediated by vascular endothelial growth factor receptor-3 (VEGFR-3) expressed on lymphatic endothelial cells (LECs) activated by its ligands VEGF-C or VEGF-D [9]. This is supported by studies demonstrating increased activities of VEGFR-3-stimulated LECs [10], correlation of overexpressed VEGF-C/-D in tumor cells with increased LVD and LN metastasis [11], and suppression of tumor lymphatic formation by inhibitors of VEGFR-3 or its ligands [12,13]. While these reports support the pro-lymphatic role of VEGFR-3, this concept does not explain well-established observations associated with lymphatic sprouting: (1) expression of LEC-specific markers VEGFR-3 [14], LYVE-1 [15], and podoplanin [16] in tumor-associated macrophages (TAMs); and (2) expression of myeloid markers CD11b [14], CD68 [17], or PU.1 [17] in tumor but not normal lymphatic vessels. The first question has been fairly well resolved by past studies that showed that myeloid cells with LEC markers are, in fact, bone marrow (BM)-derived myeloid–lymphatic endothelial cell progenitors (M-LECPs) [18]. The BM origin of myeloid–lymphatic progenitors has been shown by tracing green fluorescent protein (GFP) [14,16] and other BM-specific tags [19]. Similar cells have been shown to circulate as CD14^+^ monocytes in the blood of cancer patients [20,21], reside in mouse [15] and human [17,22] tumors, and increase tumor LVD [17] and metastasis to LNs [23]. Similar to BM progenitors to other lineages, M-LECPs express specific markers of their targeted lineage (i.e., lymphatic endothelium), which can explain their co-expression of myeloid and lymphatic markers. However, the prior studies did not explain expression of myeloid-specific markers in newly formed tumor lymphatic vessels absent from normal vasculature.

This phenomenon has been well documented in multiple tumor models including fibrosarcoma [24], insulinoma [14], melanoma [15], breast [25], colorectal [26], and prostate [14] cancers as well as at inflammatory sites in human patients [27]. This previously was attributed to insertion of myeloid cells or TAMs into remodeling lymphatic vessels [28], implying co-localization of cells of two distinct lineages. However, our prior analysis by confocal microscopy and 3D video showed intracellular expression of myeloid markers in tumor LECs [17], suggesting fusion of myeloid cells with vessels rather than macrophage transmigration through the endothelial layer or integration into the vascular wall. The fusion hypothesis is supported by two independent arguments. First, fusion is a well-known mechanism of regenerative BM-derived myeloid progenitors. This has been extensively shown through tracing myeloid and stem cell markers in hybrids generated by fusion of progenitors with damaged neurons [29], cardiomyocytes [30], skeletal muscle [31], fibroblasts [32], hepatocytes [33], and epithelial [34] and vascular endothelial [35] cells. Fusion of progenitors with injured cells induces nuclear reprogramming [36,37] which triggers re-entry of heterokaryons into the cell cycle [32] and increased cell division [34,38]. The tumor microenvironment (TME) is particularly conducive for fusion as indicated by the propensity of malignant, infiltrating, and stromal cells to create multinucleated homotypic and heterotypic hybrids [39]. Tumor-infiltrating myeloid progenitors that compose the majority of TAMs [40] are highly fusogenic as indicated by their ability to generate hybrids with neoplastic cells in glioma [41], melanoma [42], ovarian [43], lung [44], intestinal [45], and breast [46] cancers. Given that all regenerating cells use fusion to prompt division of their targeted cells, it stands to reason that M-LECPs employ the same mechanism to repair and expand lymphatics.

Fusogenicity of M-LECPs is also supported by their expression of M2 markers [17] typically associated with immunosuppressive TAMs and “wound-healing” reparative macrophages. Polarization towards the M2 phenotype is primarily regulated by Th2 cytokines interleukin-4 (IL-4), IL-13, and IL-10 [47,48]. We previously showed that Th2 cytokines induce not only M2 characteristics in differentiating M-LECPs but also lymphatic properties similar to those induced by TLR4 ligands [25,49]. Indeed, TLR4-activated myeloid precursors strongly respond to all Th2 factors due to significant upregulation of their receptors by LPS [49]. Two of these factors, IL-4 and IL-13, are the only known cytokines capable of inducing fusogenic properties in various myeloid cells including osteoclasts [50], granuloma macrophages [51], and BM-derived myeloid cells [52]. This led us to hypothesize that Th2 pathways and their upstream regulator TLR4 [49,53] promote acquisition of fusogenic properties in M-LECPs, thus enabling their merging with lymphatic vessels and subsequent transfer of myeloid markers to new LECs. To explore this hypothesis, we determined the expression of fusogenic markers in human and mouse M-LECPs as well as tested their ability to fuse with LECs in vitro and tumor lymphatic vessels in vivo. We also examined the potential of Th2 and TLR4 modulators to regulate M-LECP fusion with LECs in these settings. The rationale for this study was two-fold: to explain well-documented phenomena associated with generation of new lymphatic vessels and provide the evidence for a new mechanism driving their formation. As described below, the study results demonstrated, for the first time, that myeloid–lymphatic progenitors are fusogenic and use this ability to induce tumor LEC division. Discovery of this mechanism complements the canonical concept of VEGFR-3-driven lymphangiogenesis, thus providing a fresh perspective for development of new therapies targeting excessive or deficient function of lymphatics in a variety of human disorders.

## 2. Materials and Methods

### 2.1. Materials

Dulbecco’s low-glucose modified Eagle’s medium (DMEM) without phenol red, standard medium supplements, TrypLE, human plasma fibronectin, Dulbecco’s phosphate-buffered saline (DPBS), Image-iT FX signal enhancer, Hoechst stain, and ProLong Gold mounting medium were purchased from Thermo Fisher Scientific (Rockford, IL, USA). Fetal bovine serum (FBS) was purchased from Biowest USA (Bradenton, FL, USA). Mouse gamma globulins and lipopolysaccharide (LPS) from *E. coli* 055:B5 were from Sigma-Aldrich (St. Louis, MO, USA). A > 99% pure monospecific TLR4 inhibitor TAK-242 (cat#S7455) was from Selleckchem (Houston, TX, USA). Endotoxin-low, fatty-acid-free, >97% pure bovine serum albumin (BSA) was from MP Biomedicals (cat#0219989950, Irvine, CA, USA). Paraformaldehyde (PFA) was from Electron Microscopy Sciences (Hatfield, PA, USA). CSF-1, IL-4, and IL-10 cytokines were from BioLegend (San Diego, CA, USA).

### 2.2. Antibodies

Primary antibodies used for immunofluorescence (IF), flow cytometry, and cell fusion blocking are listed in Supplementary Table S1. Secondary antibodies were purchased from Jackson ImmunoResearch Laboratories (West Grove, PA, USA).

### 2.3. Animal Ethics Statement

Animal experiments were conducted in accordance with recommendations cited in the Guide for the Care and Use of Laboratory Animals of the National Institute of Health. Protocols #2022-110 and #2023-117 were approved by the Laboratory Animal Care and Use Committee of Southern Illinois University School of Medicine.

### 2.4. Human Blood and Tissues

Human specimens from healthy donors were purchased from the Simmons Cancer Institute Tissue Bank (Southern Illinois University School of Medicine). All samples were de-identified and collected in accordance with a protocol approved by the Springfield Committee for Research Involving Human Subjects. Blood was also purchased from Research Blood Components (Boston, MA, USA) and de-identified normal mammary tissues and BC specimens were additionally purchased from ILSBio (Baltimore, MD, USA).

### 2.5. Isolation and Differentiation of Blood-Circulating CD14^+^ Human Monocytes

Isolation of CD14^+^ monocytes was conducted using standard methods. Blood was diluted 1:2 with 2% FBS in DPBS, layered on top of Lymphoprep in SepMate tubes (StemCell Technologies, Vancouver, BC, Canada) according to the manufacturer’s instructions, and centrifuged at 1200 rpm for 1 h. Monocytes were isolated from the buffy coat using anti-CD14 IgG-conjugated magnetic beads (Miltenyi Biotec, Gaithersburg, MD, USA). Isolated cells were confirmed to be >90% viable. Cells were plated in 10% DMEM with standard supplements and human CSF-1 and IL-3 (50 ng/mL each factor). On the 9th day of culture, cells were treated with 50 ng/mL of IL-4 or IL-10. After 4 days of differentiation, cells were analyzed by RT-qPCR for expression of M2 and fusogenic markers.

### 2.6. Isolation and Differentiation of Mouse BM Cells into M-LECPs

BM cells obtained from long bones of female C57BL/6J mice (Jackson Labs, Bar Harbor, ME, USA) were crushed using a mortar and pestle in 10 mL of DPBS containing 0.5% BSA and 2 mM EDTA. After passing through a 70 µm strainer, cells were spun down at 1000 rpm for 10 min. Pellets were resuspended in 5 mL of DMEM containing 10% FBS and standard supplements and counted. Then, 10 × 10^6^ cells in 10 mL of growth medium with CSF-1 (10 ng/mL) were seeded in 10 cm^2^ dishes coated with fibronectin (10 µg/mL). After three days, attached cells were washed with DPBS and stimulated with CSF-1 combined with IL-4 or IL-10 (10 ng/mL each factor) or LPS (3 nM). On day 6, cells were analyzed for expression of M2 and fusogenic markers by flow cytometry and RT-qPCR using antibodies and primers listed in Supplementary Table S1 and Table S2, respectively.

### 2.7. RT-qPCR Analysis

RNA extraction and cDNA synthesis were performed according to the manufacturer’s instructions using RNeasy Mini and Revert Aid cDNA Synthesis kits (Thermo Fisher Scientific), respectively. Concentrations and quality of RNA and cDNA were determined by a NanoDrop2000. Primers were designed based on CDS of human or mouse targets found in the NCBI database and their sequences are listed in Supplementary Table S2. RT-qPCR of each sample was performed in duplicate using GoTaq Green Master Mix (Promega, Madison, WI, USA) and a MasterCycle Realplex PCR machine (Eppendorf, Hauppauge, NY, USA). Reaction conditions consisted of an initial denaturation step at 95 °C for 1 min followed by 38 cycles of denaturation, annealing, and extension at 95 °C, 60 °C, and 72 °C, respectively. A final melt curve was calculated by heating from 60 °C to 90 °C. Data were normalized to β-actin and relative mRNA expression was determined using the ΔΔCt method. The results are presented as the average fold change of target expression in cells differentiated by CSF-1 and LPS or Th2 factors compared with undifferentiated cells.

### 2.8. Flow Cytometry

Freshly isolated and differentiated mouse BM cells (2 × 10^5^ per sample) were incubated with gamma globulins (10 µg/mL) to block Fc receptors followed by incubation with antibodies against fusogenic markers Sirp-a, Trem2, stabilin-1 (Stab-1), DC-stamp, CD36, and CD47 (5 µg/mL). After a 1 h incubation on ice, cells were washed with ice-cold F-buffer (DPBS with 2% BSA and 0.2% of sodium azide) and incubated with 1 µg/mL of 488- or 647-conjugated secondary antibody for 1 h on ice. After washing with F-buffer, cells were fixed for 10 min with 1% PFA and analyzed using an Accuri™ C6 flow cytometer (BD Accuri Cytometers, San Jose, CA, USA) with data analyzed using FlowJo_v10 software (Tree Star, Ashland, OR, USA). Results are presented as the mean percentage of positive cells ± S.D. All experiments were reproduced twice.

### 2.9. Culture of Human and Mouse Breast Carcinoma Cell Lines

Human BC cell line MDA-MB-231 and mouse EMT6 were obtained from ATCC (Manassas, VA, USA) and engineered for stable expression of luciferase (Luc) as described previously [54]. Lines were cultured in DMEM containing 5% FBS, 2 mM of glutamine, 1 mM of sodium pyruvate, and 1 mM of non-essential amino acids at 37 °C in 10% CO_2_. Cells were passaged biweekly by incubating for five minutes at 37 °C in 0.5 mM of EDTA in DPBS followed by TrypLE. Cells were routinely tested for mycoplasma by an e-Myco Mycoplasma PCR Detection Kit (Bulldog Bio, Portsmouth, NH, USA). The MDA-MB-231-Luc line was authenticated by ATCC. EMT6-Luc cells were screened by Impact III testing through RADIL (Columbia, MO, USA) for mouse pathogens and determined to be negative.

### 2.10. Immunofluorescence

Frozen tumors were sectioned to 8 µm in thickness, fixed with acetone, and rehydrated in PBS with 0.1% Tween 20 (PBST) before incubation with the Image-iT FX signal enhancer for 30 min at room temperature. Primary antibodies diluted 1:100 in PBST containing 0.5% BSA (IHC buffer) were incubated with sections at 4 °C overnight. Slides were washed for 10 min in PBST followed by incubation with secondary antibodies diluted 1:100 in IHC buffer at 37 °C for 1 h. After a 10 min wash in PBST, sections were incubated for 5 min with 2 µg/mL of Hoechst dye. Slides were washed and mounted in ProLong Gold medium. Images were acquired using an Olympus BX41 microscope equipped with a DP70 digital camera and DP Controller NX 2.0.990.2 software (Olympus, Tokyo, Japan).

### 2.11. Quantification of Mouse Tumor Lyve-1^+^ M-LECP Expressing Fusogenic Markers

Sections from normal mouse mammary fat pads (MFPs) and MDA-MB-231 BC xenografts (*n* = 5) were co-stained with antibodies against the lymphatic marker Lyve-1 and fusogenic markers Sirp-a, CD36, DAP12, or Dc-stamp. A minimum of one hundred total Lyve-1^+^ cells were quantified per section, and the percentages of cells with and without fusogenic markers were averaged per group. Results are presented as the mean percentage of double-positives for each marker from the total analyzed Lyve-1^+^ cells.

### 2.12. Quantification of Human Tumor LYVE-1^+^ M-LECP-Expressing Fusogenic Markers

Normal and malignant human breast sections (3–5 per group) were co-stained with antibodies against LYVE-1 and fusogenic markers SIRP-A, CD36, or DAP12. A minimum of one hundred total LYVE-1^+^ cells were quantified per section, and the percentages of cells with and without fusogenic markers were averaged per group. Results are presented as the mean percentage of double-positives for each marker from total analyzed LYVE-1^+^ cells.

### 2.13. In Vitro Model for Fusion of Myeloid and Lymphatic Endothelial Cells

Rat lymphatic endothelial cells (RLECs) characterized previously [55] were transduced with lentiviral particles to constitutively express mCherry and are referred to as RLEC-mCHR. Primary BM cells from C57BL/6-Tg(UBC-GFP)/20Scha/J mice were differentiated into M-LECPs by CSF-1 followed by LPS or IL-4 or IL-10 protocols [49] and are referred to as BM-GFP. Tagged RLECs and M-LECPs were co-cultured in 12-well plates (50,000 cells of each type per well) in 1 ml of DMEM with 10% FBS (10% DMEM). After overnight incubation at 37 °C, medium was replaced with 1% BSA in DMEM. For imaging, cells were co-cultured on 0.2% gelatin-coated slides in 100 µL of 10% DMEM in a sterile chamber (20,000 cells of each type per well). After overnight incubation at 37 °C wells or slides were washed with DPBS−/− thrice and medium was replaced with 1% BSA in DMEM. Additionally, fusion assays were performed with RLEC-mCHR and the RAW264.7 macrophage line transduced with lentivirus to express GFP (termed RAW-GFP). RAW-GFP and RLEC-mCHR cells were seeded in 1 ml of DMEM containing 5% FBS at a 1:2.5 ratio in 12-well plates. After incubation overnight at 37 °C, medium was replaced with 1% BSA in DMEM with or without pro- or anti-fusogenic regulators, and fusion was monitored for 5 days. For imaging, 35,000 RLEC-mCHR cells in DMEM with 5% FBS were seeded per well of 0.2% gelatin-coated slides placed in a sterile chamber and allowed to adhere overnight at 37 °C. Slides were washed 3 times with DPBS before seeding 10,000 RAW-GFP cells in DMEM containing 1% BSA and co-cultured for 1–5 days. Co-cultures were monitored for spontaneous fusion for 5 days. In some experiments, cells were co-cultured in the presence of blocking antibodies (5 µg/mL), as specified in figure legends. TAK-242, a specific TLR4 inhibitor, was used at a concentration of 10 µM. Fusion rates were determined daily using fluorescent microscopy and flow cytometry as described below.

### 2.14. Quantification of Fusion Events In Vitro

For quantification by microscopy, images from 10–12 fields per well of 12-well plates were captured using an Olympus BX41 microscope. Total GFP^+^ and mCHR^+^ as well as double-positive cells with yellow color evident in superimposed images were enumerated and the data from triplicate wells were averaged. The results are presented as the mean percent of fused cells ± S.D. from total cells per field visualized at 400× magnification. Alternatively, dislodged co-cultured cells were resuspended in 200 µL of 10% DMEM and analyzed for dual expression of GFP and mCHR using a BD FACSAria^TM^ Flow Cytometer (BD Life Sciences, San Jose, CA, USA) and FlowJo_v10 software (Tree Star, Ashland, OR, USA). Results are presented as the mean percentage of fused cells in triplicate wells from total LECs ± S.D. All experiments were reproduced twice.

### 2.15. High-Resolution Imaging for Multinucleation

Slides were washed with 100 µL of DPBS^−^/^−^ followed by 5 min incubation in 4% PFA. Then, slides were washed twice with DPBS, incubated with 5 µg/mL of Hoechst dye, and mounted in ProLong Gold medium. High-resolution images for multinucleation were acquired using a Zeiss LSM800 confocal microscope equipped with Airyscan and analyzed with Zen Blue 3.0 software at 400× and 630× (Carl Zeiss GbmH, Jena, Germany) or an Olympus BX41 microscope.

### 2.16. Generation of Chimera Female Mice with Male Bone Marrow

Female CB-17 SCID and BALB/c mice were irradiated with 350 and 800 cGy, respectively. Twenty-four hours later, BM cells were extracted from long legs of male SCID or BALB/c mice and resuspended in DPBS containing 0.5% BSA and 2 mM EDTA. Female mice were injected intravenously with 10 × 10^6^ BM cells from male donors from the appropriate strain. Mice were monitored daily for the first week and twice a week thereafter for signs of distress. Mice surviving 30 days were considered to be fully reconstituted.

### 2.17. Orthotopic Breast Cancer Models and TAK-242 Treatment

Orthotopic BC models were previously described [54]. SCID and BALB/c female chimera mice with male BM were orthotopically implanted in the left mammary fat pad with 4 × 10^6^ MDA-MB-231-Luc or 1 × 10^6^ EMT6-Luc tumor cells suspended in 50% Matrigel, respectively. A week post-implantation, mice were randomized into two groups (*n* = 5 per group) treated either with TAK-242 dissolved in 0.5% DMSO (3 mg/kg) or vehicle alone (control group). TAK-242 and vehicle were administrated i.p. 3 times a week for the duration of the study. Tumor volume was measured every 2–3 days by determining perpendicular diameters using digital calipers and calculating the volume according to the following formula: volume = Dd^2^ π/6, where D and d are equal to larger and smaller diameters, respectively. Tumors reaching 1 cm^3^ in size were harvested along with ipsilateral and contralateral LNs and either frozen in liquid nitrogen or prepared for in situ hybridization. The latter procedure required fixation in Carnoy’s solution followed by recovery in 20% sucrose and embedding in freezing medium prior to tissue sectioning. Animal care was in accordance with institutional guidelines.

### 2.18. Measurement of Metastatic Burden

Metastatic burden in ipsilateral lymph nodes and lungs was determined as described previously [54]. Briefly, tissues were homogenized in ice-cold lysis buffer (Promega) containing protease inhibitors. Luciferase substrate (50 μL) was mixed with lysates (20 μL) followed by luminescence detection using a luminometer (Berthold, Bad Wildbad, Germany). Extracts with luciferase activity of 800 RLU/s above background were considered positive for metastases. Data are expressed as the mean RLU/s ± SEM from duplicate readings normalized per mg of total protein determined by Bradford assay.

### 2.19. Fluorescent in Situ Hybridization for Detection of Y-Chromosomes

Six-micrometer-thick tissue sections fixed by Carnoy’s solution were boiled in citric acid buffer (pH 6.0) for 20 min following by cooling to room temperature (RT) and two washes in PBS for 5 min. This was followed by a 3 min incubation in 2 × SSC buffer at RT and a 20 min incubation in 1 M sodium isothiocyanate buffer at 75 °C. Then, sections were washed twice in 2 × SCC buffer for 3 min, dehydrated through 70–90% ethanol gradients, and air dried. Biotin-conjugated Y-chromosome probe (Empire Genomics, Buffalo, NY, USA cat#MCENY-BIO-10) was diluted in supplied buffer, vortexed, and pre-warmed to 75 °C for 5 min. The warmed probe mixture was applied to the section, covered by a coverslip, and sealed with rubber cement. Sealed slides were placed in a humid chamber and incubated at 75 °C for 5 min followed by incubation at 37 °C overnight. The coverslip was removed and the slides were washed 3 times in 0.4 × SSC buffer with 0.3% NP-40. Slides were incubated with FITC-conjugated streptavidin followed by amplification with anti-FITC antibody. Tissues were co-stained for markers of lymphatic vessels (Lyve-1), blood vessels (Meca-32), M2 myeloid cells (CD206), and cell division (acetylated histone-3). After incubation with secondary antibodies, sections were incubated with 5 µg/mL of Hoechst dye and mounted in ProLong Gold medium. High-resolution images were acquired using a Zeiss LSM800 confocal microscope equipped with Airyscan and analyzed with Zen Blue 3.0 software (Carl Zeiss GbmH, Jena, Germany).

### 2.20. Quantification of Y-Chromosome-Positive Structures

To quantify lymphatic or blood vascular endothelial cells and M2 myeloid cells with nuclei-localized Y-chromosomes, 5–10 images per section were captured at 400× magnification using an Olympus BX41 microscope. The percentages of nuclei with or without Y-chromosomes were quantified in co-localized images using ImageJ 1.54p software (http://rsbweb.nih.gov/ij/, accessed on 25 May 2025).

### 2.21. Quantification of Dividing Endothelial Cells in Tumor Lymphatic and Blood Vessels

Lyve-1^+^ and Meca-32^+^ vessels were co-stained with antibody against acetylated histone-3 (Acetyl-H3) on tumor sections probed for the presence of Y-chromosomes. Vessels were enumerated and designated as either positive or negative for Y-chromosomes. Blood and lymphatic vessels with and without Y-chromosomes were quantified for expression of acetylated histone-3 (Acetyl-H3), a marker of proliferation [56]. This analysis applied to all tumors in chimera tumor-bearing mice (*n* = 5 per group). The data are presented as the mean percentage of dividing lymphatic or blood endothelial cells from total vascular structures with and without Y-chromosomes per group.

### 2.22. Statistical Analysis

Statistical significance of differences between control and experimental groups was determined by the Mann–Whitney U test (tumor growth rate), Fisher’s exact test (metastatic burden), or Student’s *t*-test (all other assays) using GraphPad Prism v7 software. *p*-value ≤ 0.05 was considered significant.

## 3. Results

### 3.1. Th2 Cytokines IL-4 and IL-10 Induce Transcripts of Fusogenic Markers in Mouse M-LECPs and Human Monocytes

**Figure 1:**
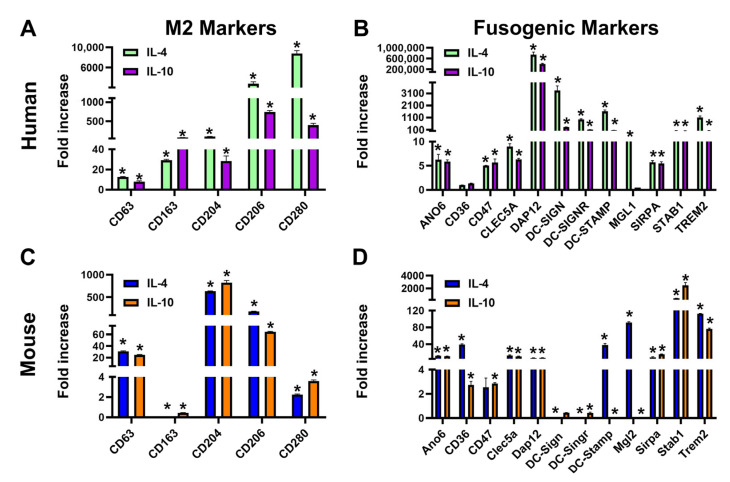
Th2 cytokines induce transcription of fusogenic markers in mouse M-LECPs and human monocytes. Human blood-circulating monocytes (**A**,**B**) or mouse BM cells (**C**,**D**) were primed with CSF-1 followed by activation with IL-4 or IL-10 as described in Methods. Transcript levels of M2 (**A**,**C**) and fusogenic (**B**,**D**) markers in freshly isolated and differentiated cells were determined by RT-qPCR and normalized for beta-actin. Results are presented as the mean fold increase in differentiated compared with control cells ± S.D. Statistical differences between control and IL-4/IL-10-differentiated cells were determined by Student’s *t*-test with *p*-values < 0.05 indicated by asterisks.

We previously showed that TLR4 and its downstream upregulated Th2 pathways induce both the immunosuppressive M2 and lymphatic phenotypes in BM-derived M-LECPs [49]. However, prior analysis did not include fusogenic mediators known to be upregulated by IL-4 or IL-13 [57]. To test the hypothesis that fusogenicity is co-acquired during Th2-induced differentiation, we compared expression of known fusogenic markers in human CD14^+^ monocytes and mouse BM cells sequentially treated with CSF-1 and IL-4 or IL-10. IL-13 has not been included in this analysis as both IL-4 and IL-13 activate a joint pathway due to a shared receptor in hematopoietic cells [58]. As expected, M2 markers were strongly upregulated by IL-4 and IL-10 in both human and mouse cells, indicating full functionality of these factors (Figure 1A,C). Additionally, Th2 factors induced transcription of multiple fusogenic mediators (Figure 1B,D). Fusogenic regulators were upregulated by Th2 cytokines by 4–100-fold and up to 1000-fold in mouse and human cells, respectively, compared with CSF-1 alone. This effect of Th2 cytokines on BM myeloid precursors is currently poorly recognized although their induction of macrophage fusogenicity is well established [57]. Despite numerical difference in fold changes between human and mouse cells, the composition of upregulated genes largely overlapped. Cells from both species significantly upregulated ANO6, known as TMEM16, phosphatidylserine (PS) transporting channel or scramblase, which is consistent with PS surface exposure required for all cell fusion processes [59,60]. Other consistently upregulated genes included well-known fusogenic regulators SIRP-a (also known as macrophage fusion receptor [61]) and its contra-receptor CD47 [62], TREM2 [63] and its adaptor DAP12 [63], DC-STAMP [64], Stab-1 [65], and MGL1/2 [66]. Some targets (e.g., DC-STAMP and MGL1/2) were preferentially induced by IL-4, the best-known inducer of fusogenicity [51,67], but many other targets were similarly increased by IL-10 (Figure 1B,D), which has not been reported previously. These data show that Th2 cytokines induce both immunosuppressive M2 and fusogenic phenotypes in human and mouse myelo-monocytic precursors.

### 3.2. TLR4 and Th2 Factors Upregulate Multiple Fusogenic Proteins During Differentiation of M-LECPs

**Figure 2:**
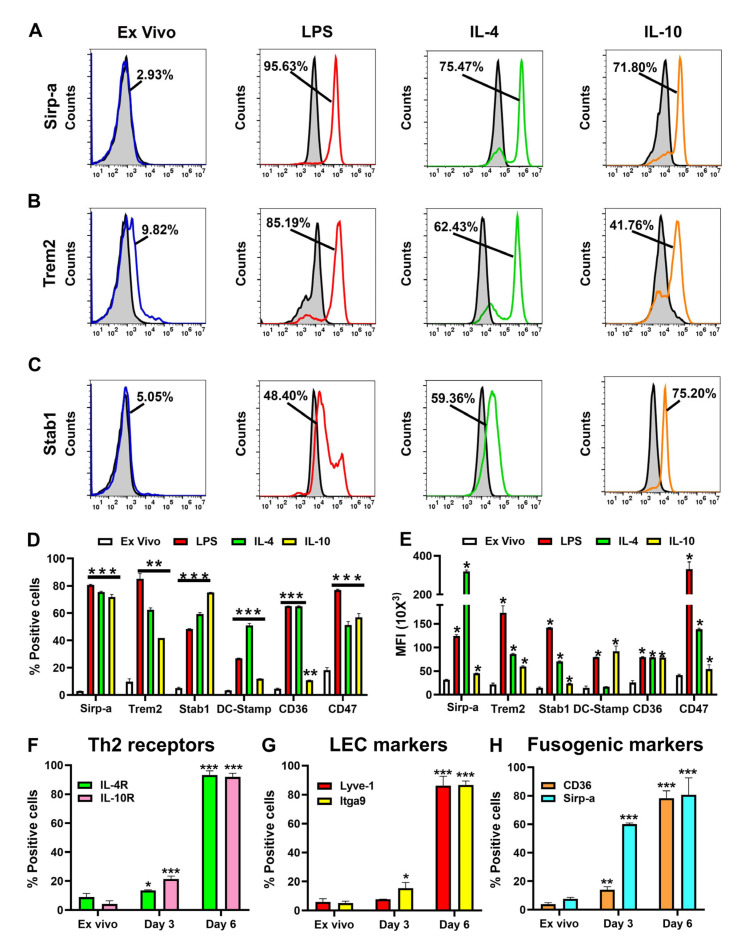
TLR4 and Th2 pathways upregulate fusogenic proteins during differentiation of M-LECPs in a time-dependent manner. Expression of fusogenic markers Sirp-a (**A**), Trem-2 (**B**), and Stab1 (**C**) was determined by flow cytometry in freshly isolated BM cells (blue lines) and cells differentiated with LPS (red line), IL-4 (green line), or IL-10 (orange line). Secondary controls in representative histograms are indicated by black lines and gray shading. The numbers in histograms indicate the mean percentage of positive cells. The mean percentage of positive cells and mean fluorescent intensity (MFI) of fusogenic markers in naïve and differentiated cells are shown in (**D**,**E**), respectively. (**F**–**H**) panels show the percentage of positive cells for Th2 receptors, LECs, and fusogenic markers on differentiation days 0, 3, and 6, respectively. All analyses were performed in duplicate and reproduced twice. Significant differences in marker expression in naïve and differentiated cells were determined by Student’s *t*-test with *p*-values < 0.05, <0.01, and <0.001 indicated by *, **, and *** symbols, respectively.

We next used flow cytometry to confirm protein expression of fusogenic genes in LPS- and Th2-factor-differentiated M-LECPs. All stimuli significantly increased both percentage of positive cells and mean fluorescent intensity (MFI) of all examined markers with *p*-values less than 0.001 (Figure 2A,B,C and Table 1). The percentages of positive cells for Sirp-a, Trem2, stabilin-1, Dc-stamp, CD36, and CD47 rose from 3–10% in ex vivo cell populations to 40–60% and 80–90% in cells differentiated by Th2 factors and LPS, respectively (Figure 2D and Table 1). The MFI values reflecting expression per cell also increased by 2- to 10-fold (Figure 2E and Table 1). Moreover, the timeframe of upregulation of fusogenic proteins largely overlapped with increased expression of Th2 receptors and LEC markers peaking on day 6 of differentiation while being low (5–10%) in ex vivo cells and only modestly increased after priming with CSF-1 on day 3 (Figure 2F,G,H). These data show that activation of TLR4 and downstream Th2 pathways in differentiating BM cells confers parallel abilities to suppress immune cells, acquire a lymphatic phenotype, and fuse with targeted cells. Coincident induction of these functions in emerging progenitors suggests they are all required for the formation of new lymphatics.

### 3.3. Multiple Fusogenic Proteins Are Expressed in M-LECPs Recruited to Mouse and Human Tumors In Vivo

**Figure 3:**
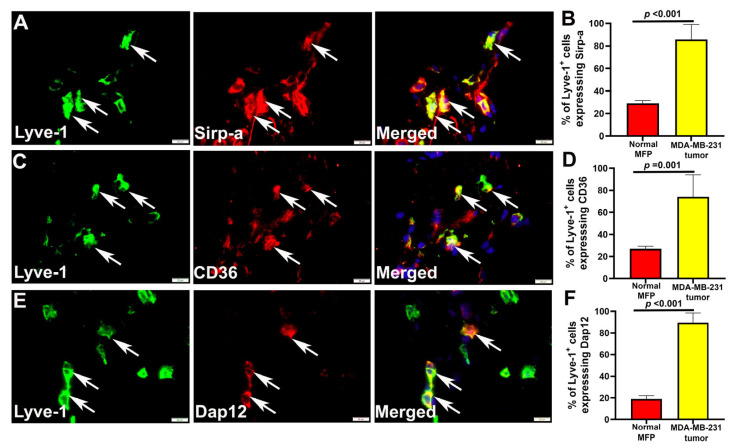
Multiple fusogenic proteins are expressed in mouse M-LECPs recruited to MDA-MB-231 tumors. Sections from normal MFPs and orthotopic tumors grown in mice (*n* = 5) were stained with antibodies against Lyve-1 and fusogenic markers Sirp-a (**A**), CD36 (**C**), and Dap12 (**E**). White arrows point to Lyve-1^+^ cells expressing fusogenic proteins. All images were acquired at 400× magnification. Quantification of Lyve-1^+^ cells that co-expressed Sirp-a (**B**), CD36 (**D**), and Dap12 (**F**) in mouse normal mammary tissues and BC xenografts was performed as described in Methods. Results are presented as the mean percent of double-positive cells as determined in a minimum of 100 Lyve-1^+^ cells per section. Differences in percentages of double-positive cells in mouse normal MFPs and MDA-MB-231 tumors were determined by Student’s *t*-test and *p*-values are listed above the black brackets.

We next examined expression of fusogenic proteins in lymphatic progenitors recruited to breast tumors in mouse models and clinical BC in vivo. For analysis of a BC model, we selected orthotopically grown MDA-MB-231 tumors well known to recruit M-LECPs, induce lymphatic vessel sprouting, and metastasize to LNs [68,69]. Normal mammary fat pads (MFPs) and tumor sections (*n* = 5 per group) were double-stained for Lyve-1 to identify single M-LECP cells and eight proteins known to promote cell fusion including CD36 [57], CD98 [70], CD209 [71], Dap12 [63], Dc-stamp [63], Sirp-a [72], Sirp-b1 [73], and stabilin-1 [65]. All eight markers were detected in 73–98% of intratumoral Lyve-1^+^ cells in all analyzed tumors (Figure 3A,C,E, Table 2). In contrast, MFPs from tumor-free mice contained only 20–28% marker-positive Lyve-1^+^ cells (Figure 3B,D,F). Expression of multiple fusogenic proteins in the majority of tumor M-LECPs (Table 2) supports the necessity of this function for expansion of lymphatics. Significantly lower (*p*-values < 0.001) but noticeable expression of fusogenic proteins in M-LECPs in tumor-free mice suggests that steady-state maintenance of lymphatic vessels might also be mediated through fusion with progenitors.

**Figure 4:**
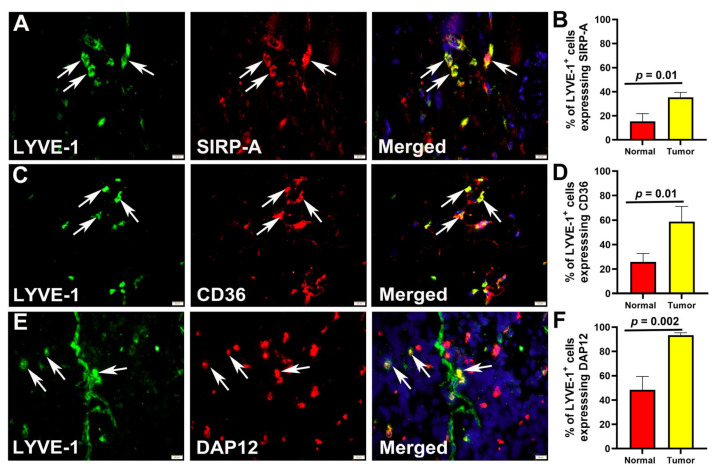
Human tumor M-LECPs expressing fusogenic proteins. Sections of clinical breast tumors (*n* = 3) were co-stained with antibodies against lymphatic marker LYVE-1 and fusogenic markers SIRP-a (**A**), CD36 (**C**), and DAP12 (**E**). White arrows point to LYVE-1^+^ cells expressing fusogenic proteins. All images were acquired at 400× magnification. Quantification of LYVE-1^+^ cells that co-expressed SIRP-a (**B**), CD36 (**D**), and DAP12 (**F**) in human normal mammary tissues and breast tumors was performed as described in Methods. Results are presented as the mean percentage of double-positive cells as determined in a minimum of 100 LYVE-1^+^ cells per section. Differences in percentages of double-positive cells in human normal and malignant breast tissues were determined by Student’s *t*-test and *p*-values are listed above the black brackets.

This conclusion was further validated using human clinical BC specimens and normal mammary tissues. Sections were double-stained with antibodies against LYVE-1 and some of the most prominent fusogenic markers such as SIRP-a, CD36, and DAP12 (Figure 4A,C,E). The mean percentages of tumor M-LECPs co-expressing SIRP-a, CD36, and DAP12 were 35.26 ± 4.13, 58.81 ± 12.33, and 93.36 ± 1.99, respectively (Figure 4B,D,F).

As in mouse-grown tumors, these values were 2- to 2.5-fold higher than the percentage of double-positive M-LECPs in normal specimens (Supplementary Figure S1, *p*-values < 0.01). Collectively, these data show for the first time that mouse and human tumor-recruited M-LECPs have high fusogenic potential that likely is pre-determined along with immunosuppressive and lineage-specific traits during differentiation in the BM (Figure 1 and Figure 2).

### 3.4. BM-Derived M-LECPs and RAW264.7 Macrophages Fuse with Lymphatic Endothelial Cells In Vitro

**Figure 5:**
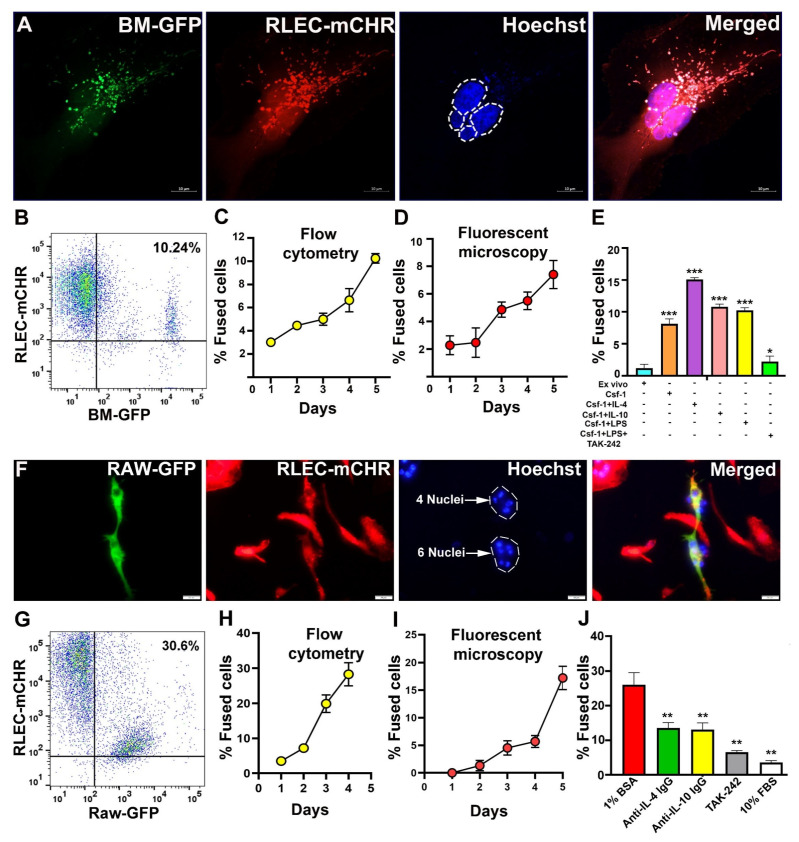
Primary M-LECPs and RAW264.7 macrophages fuse with LECs in vitro. (**A**,**F**) Representative images of M-LECPs from BM of GFP^+^ mice (**A**) or GFP-expressing RAW264.7 cells (**F**) co-cultured with RLEC-mCherry (mCHR) for 5 days in 1% BSA medium. Hoechst-stained multiple nuclei are highlighted by white circles. Images were acquired at 400× magnification. (**B**,**G**) Flow cytometry analysis of M-LECP (**B**) and RAW264.7 (**G**) double-positive cells co-expressing GFP and mCherry on the fifth day of co-culture. Numbers in upper right quadrants indicate percentages of fused cells. (**C**,**H**) Daily quantification of fusion mediated by M-LECPs (**C**) or RAW264.7 cells (**H**) using flow cytometry. (**D**,**I**) Daily quantification of fusion mediated by M-LECPs (**D**) or RAW264.7 cells (**I**) using fluorescent microscopy. (**E**) Flow cytometry quantification of either fused ex vivo BM-GFP cells, post-CSF-1 treatment alone, or additionally differentiated with IL-4, IL-10, or LPS in the absence or presence of the TLR4 inhibitor TAK-242. (**J**). Flow cytometry quantification of fused RAW264.7 cells with LECs in the presence of anti-IL-4 IgG, anti-IL-10 antibody (5 µg/mL each), or TAK-242 (10 µM). Here, 1% BSA and 10% FBS media were used as a positive and a negative control, respectively. All experiments were performed in duplicate and reproduced at least three times. Statistical analyses determined by Student’s *t*-test yielded *p*-values < 0.05, <0.01, and <0.001 as indicated correspondingly by *, **, and *** symbols.

We next sought to determine whether primary M-LECPs and RAW264.7 macrophages, cells that partly mimic M-LECP [18], are capable of fusing with LECs in vitro. To this end, we established a novel in vitro system [17] consisting of co-culture of M-LECPs derived from BM of GFP^+^ mice (BM-GFP) or GFP-tagged RAW264.7 macrophages (RAW-GFP) with mCherry-tagged rat LECs (RLEC-mCHR) characterized previously [55]. Fusion was detected by two independent methods, flow cytometry and fluorescent microscopy. Both methods readily detected yellow cells resulting from merging of green and red fluorescence. Less than 2% hybrids were detected by either method under standard growth conditions in the medium with 10% DMEM. However, in serum-depleted medium (1% BSA) mimicking the stress of the TME, both primary M-LECPs and RAW-GFP generated 10.2% and 30.6% yellow cells, respectively (Figure 5A,B,F,G). Fusion mediated by M-LECPs and RAW-GFP cells was associated with multinucleation indicating induction of cell division (Figure 5A,F). Both detection methods showed a time-dependent increase in fused cells in days 1–5 post-switching to serum-depleted conditions (Figure 5C,D,H,I). Primary BM cells were most fusogenic after differentiation with CSF-1 and LPS or Th2 cytokines whereas fusion was significantly reduced in naïve BM cells and those treated with TLR4 inhibitor TAK-242 [75] or 10% serum (Figure 5E).

In contrast to primary cells, neither LPS nor Th2 cytokines increased fusogenic potential of RAW-GFP cells; however, addition of blocking anti-IL-4 or anti-IL-10 antibodies or TAK-242 significantly suppressed fusion (Figure 5J). These data suggest that immortalized RAW264.7 cells have constitutively active TLR4 which leads to expression of Th2 cytokines ultimately resulting in a substantially higher fusion rate than primary differentiated BM cells (30% vs. 10%, Figure 5E,J). Robustness of this model is indicated by the ability to regulate fusion in primary BM cells and RAW264.7 cells by activation and blockade of Th2 pathways, respectively, as well as by 70–80% inhibition of TLR4 signaling (Figure 5J). Collectively, these data show that both primary M-LECPs induced by either TLR4 or Th2 pathways as well as pre-activated RAW264.7 macrophages are capable of fusing with LECs under stress conditions similar to the TME.

### 3.5. M-LECPs Recruited to EMT6 and MDA-MB-231 Tumors Fuse with Lymphatic Vessels In Vivo

**Figure 6:**
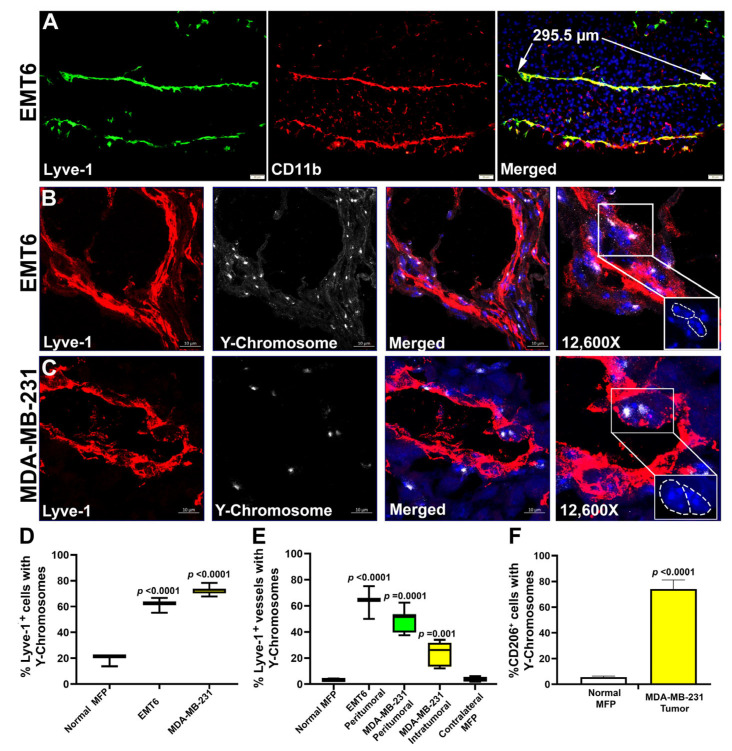
M-LECPs recruited to EMT6 and MDA-MB-231 tumors fuse with lymphatic vessels in vivo. Female BALB/c or CB-17 SCID mice were lethally irradiated and grafted with male BM from the same strain. After full reconstitution, mice were orthotopically implanted with mouse EMT6 (**A**,**B**) or human MDA-MB-231 (**C**) breast tumor cells. At the end of the experiment, tumors were analyzed for CD11b expression (**A**) or nucleus-localized Y-chromosomes in lymphatic vessels (**B**,**C**) using immunostaining and fluorescent in situ hybridization (FISH), respectively. Slides were counterstained with Hoechst dye (blue) to identify Y-chromosome nuclear localization. Z-stack images of Lyve-1^+^ vessels in EMT6 (**B**) and MDA-MB-231 (**C**) tumors acquired by confocal microscopy were captured at 630× magnification, with the final panel showing 12,600× enhancement of Y-chromosome^+^ nuclei outlined by the upper white boxes. The lower white boxes show multinucleation in the same vessels highlighted by a dotted white line. (**D**) Lyve-1 and Y-chromosomes were detected in normal MFPs, EMT6, and MDA-MB-231 tumors. Data are presented as the percentage double-positive cells out of total Lyve-1^+^ cells per field. (**E**) Percent of Lyve-1^+^ vessels with Y-chromosomes was determined in tumor-free and contralateral MFPs from tumor-bearing mice as well as in peritumoral or intratumoral regions of EMT6 and MDA-MB-231 tumors. *p*-values determined by Student’s *t*-test show significant differences between normal and tumor-containing MFPs but not contralateral MFPs from tumor-bearing mice. (**F**) CD206 and Y-chromosomes were detected in sections of normal and tumor-containing MFPs using immunostaining and FISH, respectively. Data are presented as percent of CD206^+^/Y-chromosome^+^ double-positive cells out of total CD206^+^ cells per field. All analyses were performed in three individual tumors per group using as minimum 10 fields per section.

Previous reports showed that tumor or inflamed (but not normal) lymphatic vessels tend to express myeloid-specific markers [76] which was attributed mainly to co-localization of two adjacent cell types under pathological conditions. However, we previously showed in clinical human BC through Z-stack confocal images and a 360° rotation video that myeloid-specific markers are detected within multiple LEC compartments and present throughout the endothelial layer [17]. These morphological observations suggested merging of myeloid cells with LECs rather than close interaction of two distinct cell types. This is supported here by Figure 6A that shows a 295 µm segment of a lymphatic vessel in EMT6 tumor with full overlap between Lyve-1 and CD11b staining. Since cell diameters do not exceed 20–25 µm, the most plausible explanation for co-staining is fusion of myeloid progenitors with LECs as hybrids are known to express markers of both parental cells [77].

To confirm this conclusion, we employed a standard method of tracing BM-specific markers in chimera mice. As described in Methods, native BM in female BALB/c and SCID mice was obliterated by irradiation and replaced by male BM from the same strain. After full reconstitution, mice were implanted with syngeneic EMT6 or human MDA-MB-231 tumors. Detection of Y-chromosomes in lymphatic vessels was achieved through combination of staining for Lyve-1 and FISH using a probe for mouse Y-chromosomes (Figure 6B,C). In the EMT6 model, 60.44 ± 3.25% and 63.19 ± 8.88% of total Lyve-1^+^ cells and peritumoral lymphatic vessels contained Y-chromosomes, respectively, whereas the corresponding numbers in the MDA-MB-231 model were 72.55 ± 2.72% and 48.68 ± 8.65 (Figure 6D). Highly metastatic MDA-MB-231 tumors have additional intratumoral lymphatic vessels, a quarter of which were positive for Y-chromosomes (24.17 ± 8.57%, Figure 6E). Normal and contralateral MFPs in tumor-bearing mice contained significantly lower levels of BM-derived Lyve-1^+^ cells (~20%) and hybrid lymphatic vessels (2–3%) compared with tumor counterparts (*p*-values < 0.01, Figure 6E).

Since Lyve-1^+^ cells are a subset of M2-TAMs [17], we were also interested in determining the BM origin of the total TAM population by co-staining for Y-chromosomes and CD206. The latter is an M2-specific marker upregulated in human and mouse M-LECPs by Th2 cytokines (Figure 1) known to be uniformly expressed in immunosuppressive M2-TAMs [78] along with CD11b [79]. The results show overwhelming nuclear presence of Y-chromosomes in ~80% of tumor CD206^+^ cells as opposed to 2–3% of macrophages in normal MFPs (Supplementary Figure S2; Figure 6F). Collectively, these data show that the majority of M2-type TAMs in BC models including fusogenic Lyve-1^+^ myeloid cells (Figure 1, Figure 2, Figure 3, Figure 4 and Figure 5) are derived from the bone marrow and that a substantial portion of tumor lymphatic vessels undergo fusion.

### 3.6. Fusion of Tumor Lymphatics with BM Progenitors Correlates with Increased LEC Proliferation

**Figure 7:**
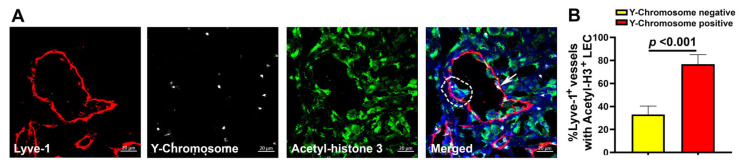
Fusion of lymphatic vessels with BM progenitors correlates with LEC proliferation. Female CB-17 SCID mice were lethally irradiated and grafted with male BM from the same strain. After full reconstitution, mice were orthotopically implanted with MDA-MB-231 tumor cells. Tumor sections were analyzed by FISH for the nuclear localization of Y-chromosomes in Lyve-1^+^ vessels. Sections were co-stained for acetyl-histone 3 (Acetyl-H3), a marker of cell division, and counterstained with Hoechst dye (blue) to identify Y-chromosomes. (**A**) Z-stack images acquired by confocal microscopy at 400× magnification demonstrate expression of Acetyl-H3 in tumor LECs. White arrow in the merged image shows nuclear detection of Y-chromosome in Lyve-1^+^ tumor lymphatic vessels. White dotted line on the opposite side of the vessel highlights multiple nuclei with a mitotic marker Acetyl-H3 and integrated Y-chromosome (white dot) signifying fusion with BM progenitors. (**B**) Quantification of Acetyl-H3 expression in Y-chromosome-negative and -positive tumor lymphatic vessels. The results are presented as the percentage of Lyve-1^+^ vessels expressing Acetyl-H3 marker in Y-chromosome^+^-positive and -negative groups. The *p*-value determined by Student’s *t*-test indicates significant difference in mitotic marker expression between the two groups of tumor lymphatic vessels.

The well-known consequences of M2-TAM fusion with tumor cells are substantially increased proliferation [80], migration [81], and survival [82] of hybrid cells. The same processes are activated in endothelial cells during the formation of new vessels [83]. We therefore hypothesized that Y-chromosome^+^ LECs might have a higher division rate than those that lacked a fusion marker. To test this hypothesis, we established a new protocol of immunostaining that included detection of a mitotic marker in addition to Lyve-1 and FISH. A choice of using an antibody against acetylated histone-3 (Acetyl-H3), a well-documented marker of cell division [56], in lieu of more conventional Ki-67 or PCNA antibodies resulted from extensive screening on tissues fixed with Carnoy’s solution, a required step for FISH detection of Y-chromosomes. Using this procedure combined with Z-stack images acquired by confocal microscopy, we quantified tumor lymphatic vessels with and without Y-chromosomes and a mitotic marker. Representative images in Figure 7A show high incidence of Y-chromosome (Y^+^) integration in the nuclei of Lyve-1^+^ vessels (four out of four in this field) with an average of 49.5 ± 6.4 of Y^+^/Lyve-1^+^ structures from the total. The largest lymphatic vessel in the shown field not only displayed multiple fusion events throughout the endothelial layer (shown by white dots) but also a cluster of multinucleated LECs that coincided with both expression of Acetyl-H3 (green) and a nucleus-localized (blue) Y-chromosome (Figure 7A, merged, white dotted line). Three out of four shown Y^+^/Lyve-1^+^ vessels (75%) also displayed nuclear expression of Acetyl-H3, which was consistent with the mean percentage of 76.78 ± 8.2% proliferating lymphatic vessels identified in all examined specimens (Figure 7B). In contrast, among Lyve-1^+^ vessels without Y-chromosomes only 33% were labeled by this mitotic marker (*p*-value < 0.001). These findings support the concept that M-LECP fusion with LECs promotes their division.

### 3.7. Fusion of Tumor Blood Vessels with BM Progenitors Also Correlates with Endothelial Cell Division

**Figure 8:**
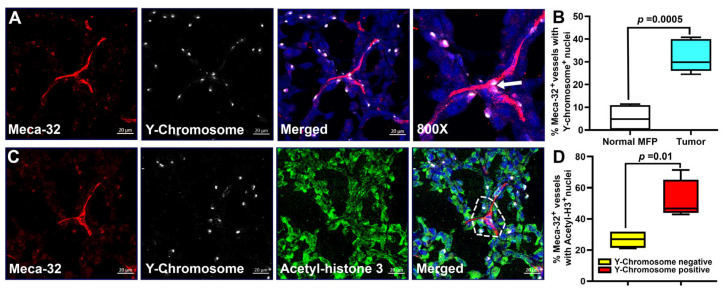
BM-derived blood vascular progenitors merged with tumor blood vessels. Female CB-17 SCID mice were lethally irradiated and grafted with male BM from the same strain. After full reconstitution, mice (*n* = 3) were orthotopically implanted with MDA-MB-231 tumor cells. (**A**) Representative images of tumors co-stained for Meca-32 (red) and Y-chromosomes (white). Slides were counterstained with Hoechst dye (blue) to identify Y-chromosome nuclear localization. Z-stack images of Meca-32^+^ vessels were acquired by confocal microscopy at 400× with the final panel showing 800× magnification. White arrow highlights vessel-integrated Y-chromosome at the fork of an expanding vascular structure. (**B**) Y-chromosome-positive blood vessels were quantified in normal and tumor-containing MFPs. The *p*-value determined by Student’s *t*-test indicates significant difference in Y-chromosome detection in blood vessels located in normal and tumor-containing MFPs. (**C**) Tumors were also co-stained for Acetyl-H3 expression to determine correlation with mitotic division. Representative images show nuclear co-localization of Acetyl-H3 and Y-chromosomes in Meca-32^+^ tumor blood vessels. (**D**) Quantification of Acetyl-H3 expression in Y-chromosome-negative and -positive tumor Meca-32^+^ vessels. The results are presented as the percentage of Meca-32^+^ vessels expressing Acetyl-H3 marker in Y-chromosome-negative and -positive groups. The *p*-value determined by Student’s *t*-test indicates significant difference in mitotic marker expression between the two groups of tumor blood vessels.

Prior studies suggested that fusion of BM-derived M2 endothelial progenitors promoted vascular repair in ischemic tissues [35]; however, no study to date has shown evidence for fusion in the context of tumor blood vasculature. Chimera mice used in this study provided an opportunity to do so. We found that up to 40% of Meca-32^+^ blood vessels in MDA-MB-231 tumors contained nuclear Y-chromosomes compared with <10% in tumor-free MFPs (Figure 8A,B)). As shown in Figure 8A, some vessels displayed nucleus-integrated Y-chromosomes in endothelial cells located at the fork of new sprouts (white arrow), suggesting a direct link between fusion and expansion of blood vasculature. The density of Y^+^/Meca-32^+^ vessels in tumors was significantly higher than that in normal MFPs (*p*-value < 0.001) which replicates the findings with lymphatic vessels (Figure 6). The similarity to lymphatic formation was further supported by high correspondence of the cell division marker Acetyl-H3 (green) with multinucleation (blue) and nuclear Y-chromosomes (white) in Meca-32^+^ blood vessels (red) (Figure 8C). In line with the data showing inclination to cell division of fused LECs (Figure 7), Acetyl-H3 was expressed in ~60% of fused Y^+^/Meca-32^+^ as compared with ~25% of cells lacking the fusion marker (Figure 8D). Taken together with findings for lymphatics, these data demonstrate that both types of vasculature, at least in part, are generated due to fusion with BM progenitors, a mechanism that has not been previously considered for either blood or lymphatic tumor vessel formation.

### 3.8. Inhibition of TLR4 Reduces Fusion of BM Progenitors with Tumor Lymphatics, LVD, and Lymph Node Metastasis

We lastly sought to determine whether inhibition of TLR4 affects fusion-generated lymphatics. Activation of TLR4 is known to promote lymphangiogenesis [84] and its absence correlates with deficiency in lymphatic vessels [85]. Consistent with that, we previously showed that TLR4 activation in BM myeloid precursors promotes differentiation of pro-lymphatic progenitors either directly [25,86] or through induction of Th2 pathways [49]. Data in Figure 1, Figure 2, Figure 3, Figure 4 and Figure 5 show that changes in either TLR4 or Th2 ligands correlate with expression of fusogenic proteins and activity. Based on this evidence, we hypothesized that inhibition of TLR4 in tumor-bearing mice would suppress fusogenicity of M-LECPs, leading to reduction in new vessels and decreased node metastasis.

**Figure 9:**
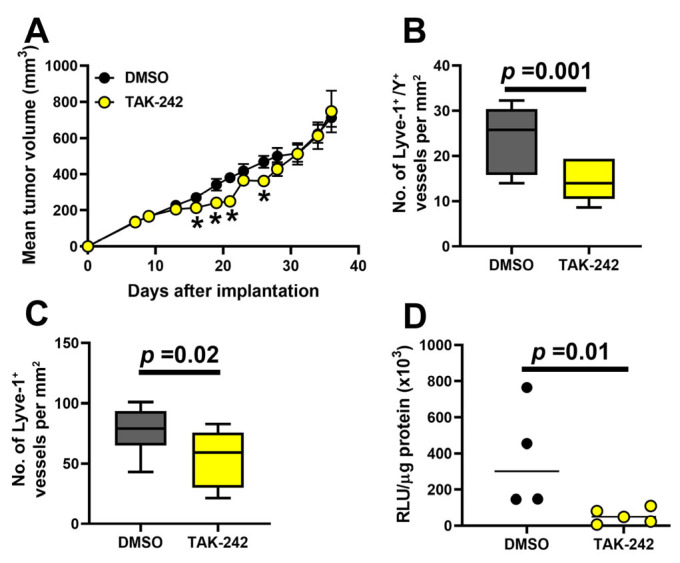
Blocking TLR4 signaling reduces M-LECP fusion, lymphatic formation, and lymph node metastasis. Female CB-17 SCID mice were lethally irradiated and grafted with male BM from the same strain. After full reconstitution, mice were orthotopically implanted with MDA-MB-231 tumor cells. Mice were treated with 0.5% DMSO vehicle control or TAK-242 (5 mice per group). (**A**) Mean tumor volumes per group treated with vehicle or TAK-242. Asterisks indicate statistically significant but transient reduction in tumor growth rate in the TAK-242-treated versus control group, as determined by Student’s *t*-test (*p* < 0.05). (**B**) The density of fused lymphatic vessels was determined in 10 fields per section at 400× magnification and normalized per mm^2^. (**C**) The area-normalized density of tumor Lyve-1^+^ vessels in control and TAK-242-treated mice was determined as described for (**B**). (**D**) Metastatic burden was determined by measuring luciferase activity in tumor-adjacent lymph nodes. Data are presented as the mean relative luciferase units (RLU) normalized per mg of protein. Statistical significance of differences between control and TAK-242 groups was determined by Student’s *t*-test and is indicated by the *p*-values listed above the black bars.

To test this hypothesis, we treated MDA-MB-231-bearing chimera female mice with male BM with 0.5% DMSO control or TAK-242 (3 mg/kg), a specific TLR4 peptide inhibitor that prevents receptor dimerization [75]. Tumors and regional LNs were collected at a tumor size of ~1 cm^3^ followed by detection of Y-chromosome^+^ fused LECs, total LV density, and nodal metastases. While the TAK-242 treatment did not have a sustainable effect on tumor growth (Figure 9A), it reduced the density of lymphatic vessels displaying a Y-chromosome fusion marker by 40.7% from 24.3 ± 7.03 to 14.4 ± 4.09 lymphatic vessels per mm^2^ in control and treated mice, respectively (*p*-value = 0.001, Figure 9B). This corresponded to reduced lymphatic density (Figure 9C) and a highly significant decrease of 85.8% in node metastatic burden (Figure 9D). The latter is shown by a decrease from 378,434 to 53,827 RLU/mg protein (*p*-value = 0.01) in control and TAK-242-treated mice, respectively. These data show that inhibition of TLR4 signaling can significantly interfere with fusion of progenitors with tumor LECs, leading to suppression of lymphatic formation and tumor spread.

## 4. Discussion

The key conclusion from this study is that myeloid–lymphatic progenitors express fusogenic markers and fuse with LECs in vitro and in vivo, leading to increased LEC division and vascular density. A similar portion of tumor blood vessels also underwent fusion and subsequently increased proliferation of the hybrid cells. We found that fusogenic properties of M-LECPs are regulated by TLR4 and downstream Th2 ligands as shown by corresponding changes in the fusion rate of M-LECPs induced by activators and inhibitors of these pathways. The fusion of M-LECPs with tumor lymphatic vessels has a significant effect on vascular expansion and LN metastasis as shown in MDA-MB-231-tumor-bearing mice treated with a blocker of TLR4. These findings show that fusion mediated by BM-derived myeloid–endothelial progenitors promotes outgrowth of tumor vessels and, by extension, metastasis.

### 4.1. BM-Derived Myeloid–Lymphatic Progenitors Are Fusogenic and Use Fusion to Induce Vascular Sprouting 

Three lines of evidence demonstrate fusogenicity of M-LECPs, and in a more limited fashion, blood vascular endothelial progenitors. First, differentiation of M-LECPs induced by LPS or Th2 cytokines upregulated multiple fusogenic markers in both mouse and human myeloid precursors (Figure 1 and Figure 2). Tumor-recruited M-LECPs retained this profile in vivo as shown by expression of prominent fusogenic markers SIRP-a, CD36, and DAP12 in 60–90% of LYVE-1^+^ cells in mouse and human BC (Figure 3 and Figure 4). Second, M-LECPs and RAW264.7 macrophages that partly mimic these progenitors [18] fuse with cultured LECs, as quantified by two independent methods (Figure 5). The fusion assay showed that naive BM cells largely lacked fusogenic potential whereas differentiated M-LECPs were highly sensitive to TLR4 and Th2 inhibitors (Figure 5E,J). Third, tracing Y-chromosomes in chimera mice confirmed the high frequency of fusion in two BC models in vivo (Figure 6 and Figure 7). Endothelial–progenitor fusion led to cell division and vascular sprouting and all these events in LECs regulated by TLR4 (Figure 8 and Figure 9). Collectively, these data show that fusion of BM pro-vascular progenitors with pre-existing inflamed endothelium triggers expansion of vasculature.

This conclusion is supported by ample evidence demonstrating fusogenic activity of other BM progenitors. Physiologically, multicellular organisms restrict fusion to: (1) fertilization [87]; (2) syncytia formation in placenta [88], muscle fibers [89], osteoclasts [90], and granulomatous macrophages [91]; and (3) tissue repair associated with wound healing [92] and cancers [37,93]. The latter is exclusively mediated by BM-derived myeloid progenitors known to be massively recruited to tumors [94]. This is consistent with our data that show that >80% of CD206^+^ TAMs carry Y-chromosomes, indicating their BM origin (Figure 6F). This finding shows that the majority of TAMs are not terminally differentiated resident macrophages but freshly recruited BM myeloid progenitors like M-LECPs.

Fusogenicity of myeloid progenitors has been shown by numerous studies demonstrating their BM origin, high fusogenic potential, and specificity of cell partners. Tracing BM-specific tags like GFP and sex chromosomes identified fusion of myeloid progenitors with injured fibroblasts [32], hepatocytes [95], cardiomyocytes [96], spinal cord neurons [97], smooth muscles [98], and epithelial cells [32,35]. Rampant fusion in tumors is well documented in studies of hybrids resulting from merging of tumor cells with M2-TAMs [80,93], most of which are BM-derived myeloid cells [94]. Although fusogenic activity of myeloid–lymphatic progenitors has not been suggested previously, it could be deduced from prior studies demonstrating persistent expression of BM-derived GFP [24,26] or myeloid markers [99] in tumor and inflamed lymphatics. This previously was characterized as integration [27,99] or incorporation [100] of myeloid cells into the vessels implying spatial co-localization of macrophages and LECs rather than fusion. However, we previously showed that myeloid-specific proteins CD14, CD68, and PU-1 are intracellularly expressed in LYVE-1^+^ tumor vessels [17], suggesting their full merging with myeloid cells. This is corroborated here by demonstration of a ~300 µm segment of a lymphatic vessel positive for CD11b (Figure 6A), which cannot be explained by insertion of single cells with a 20 to 25 µm diameter.

Fusogenicity of BM progenitors has also been shown by nuclear detection of gender-mismatched chromosomes in human or animal recipients of BM transplants (BMTs). Analysis of human cancers developed 8–10 years after BMT showed ~5% of tumor blood vessels expressing chromosomes of the opposite sex [101]. Y-chromosomes were also detected in 68% of CD31^+^ blood vessels of female-donor-derived liver tissue grafted in male recipients that contained myeloid cells [95]. Up to 13% of tumor lymphatic vessels harbored Y-chromosomes in female patients with breast or colorectal cancers who received male BMT [27]. In line with these studies, we found nuclear localization of Y-chromosomes in nearly half and a third of tumor lymphatic and blood vessels, respectively (Figure 6 and Figure 8). These values are somewhat higher than 2–25% of vessel-integrated progenitors reported in animal studies [102], but the percentage might depend on the model and quantification methods. Taking our data and evidence from independent studies together, we concluded that: (1) fusogenicity of M-LECPs is consistent with fusogenic activities reported for progenitors to other lineages; (2) fusion occurs in a sizable portion of tumor vessels; and (3) fusion significantly correlates with endothelial cell division suggesting that it is likely required for tumor vessel outgrowth.

Although some studies suggested that the pro-lymphatic role of progenitors can be explained only by over-expression of soluble factors like VEGF-C [20], fusion of progenitors with targeted cells presents distinct benefits for structural expansion. Soluble factors might have off-cell target effects and weaker responses due to reliance on intracellular mediators. In contrast, cell–cell fusion has exquisite specificity for delivering a pro-mitotic message directly to the nucleus of targeted cells, forcing them to enter the cell cycle [32,34]. This is supported by our data that show preferential expression of a mitotic marker, acetylated histone-3, in Y-chromosome^+^ endothelial cells compared with vessels lacking this marker (Figure 7 and Figure 8). Fusion-dependent reprogramming also promotes cell migration [45], survival [38], and acquisition of novel properties [77], all of which additionally benefit vascular formation.

### 4.2. Fusogenicity of Myeloid–Lymphatic Progenitors Is Regulated by CSF1-TLR4-Th2 Pathway Axis

Whereas progenitors’ use of fusion for cell renewal is well documented, little is known about inducers of their fusogenicity. The best-studied promoters of fusogenic potential in myeloid [57] and non-hematopoietic [89] cells are Th2 cytokines IL-4 and IL-13. In hematopoietic cells, these proteins share a joint signaling pathway mediated by IL-4 receptor alpha [58]. IL-4 upregulates multiple fusogenic regulators including SIRP-a [103], its counter-receptor CD47 [104], CD36 [57], DC-STAMP [63,103], CD209 [71], CCL-2 [52], stabilin [15], TREM-1/-2 [57], and its adaptor DAP12 [63]. Nearly all these proteins were detected in CSF-1 primed myeloid precursors activated by TLR4 or Th2 ligands in vitro (Figure 1 and Figure 2) and in tumor-recruited M-LECPs in vivo (Figure 3 and Figure 4, Table 1). These proteins contribute to fusogenic capacity of osteoclasts [105], monocytes [51], granuloma macrophages [106], M2-TAMs [57], and muscle precursors [89]. IL-4, for instance, doubles the rate of fusion between human monocytes and tumor cells [44]. Blockade or deletion of IL-4 or IL-13 inhibits fusion mediated by M2 macrophages [107] and muscle cell precursors [108]. In line with this evidence, we show that IL-4 significantly increased fusogenic capacity of M-LECPs whereas anti-IL-4 antibody inhibited fusion of pre-activated RAW264.7 cells (Figure 5). These data indicate that the IL-4/IL-13 pathway plays a direct regulatory role in M-LECP fusogenicity.

In contrast to known pro-fusogenic effects of the IL-4/IL-13 pathway, IL-10 was first reported as lacking this activity [109]. However, IL-10 knockout mice are deficient in generation of multinucleated osteoclasts [110] and muscle fibers [111] which leads, respectively, to bone and muscle loss. These observations suggested that IL-10 has a role in driving fusogenic potential in BM progenitors. Our data are in line with this conclusion as we show that IL-10, similarly to LPS and IL-4, upregulates multiple fusogenic markers in both human and mouse myeloid cells (Figure 1 and Figure 2). Moreover, IL-10-differentiated M-LECPs had a significantly higher fusogenic activity compared with ex vivo cells while anti-IL-10 antibody inhibited fusion of RAW264.7 cells in vitro (Figure 5). These data identified a novel role for IL-10 in induction of M-LECP fusogenicity which is acquired in the same timeframe as their lymphatic phenotype [49]. The co-expression of lymphatic and fusogenic markers has been noted for all inducers of M-LECP differentiation suggesting that both phenotypes are required for triggering lymphatic outgrowth. However, the function of lymphatic proteins expressed in these progenitors is currently unknown.

Both CSF-1 and TLR4 pathways are intimately linked to induction of Th2 cytokines responsible for the immunosuppressive phenotype of M-LECPs [17] acquired along with lymphatic markers [49] and the fusogenic potential shown here. CSF-1 as a key regulator of the myeloid lineage primes BM cells to efficiently respond to TLR4-activating signals [112]. We showed previously that sequential application of CSF-1 followed by TLR4 ligands yields functional M-LECPs able to increase lymphatics in vivo [25]. The following study showed that activation of TLR4 in CSF-1-primed BM cells strongly upregulates Th2 receptors [49], making the cells highly responsive to Th2 factors. This establishes the cascade in which regulation of each of these pathways can impact generation of M-LECPs and their effects on lymphatics. This explains, for instance, why CSF-1 knockout mice [113] and those treated with a CSF-1 inhibitor [17] have significantly reduced densities of tumor Lyve-1^+^ cells and lymphatic vessels. This is also consistent with TLR4-dependent regulation of M-LECP fusogenicity in vitro and in vivo which ultimately translates into increased lymphatic density and metastasis (Figure 5 and Figure 9).

While our data identify TLR4 expressed in BM myeloid cells as the main regulator of Th2 pathways, this receptor is best known for induction of stimulatory Th1 cytokines [114]. However, in the context of chronic or unresolved inflammation marked by elevated CSF-1, the TLR4 pathway induces Th2 cytokines to curb excessive stimulation. This is mainly mediated by transcription factor c-Maf as shown in a variety of LPS-treated myeloid cells [115,116] including RAW264.7 macrophages [18]. The shift from a stimulatory to tolerant state is regulated by transition from MyD88 to TRIF [117] and TRAM [53] adaptors that favor transcription of IL-4 and IL-10 [53,117]. Consistent with that, blocking IL-10 during differentiation of M-LECP downregulates these adaptors of TLR4, leading to reduced expression of c-Maf, Th2 receptors, and their downstream targets representing the M2 phenotype [49]. Collectively, this evidence suggests that the CSF1-TLR4-Th2 axis co-regulates M2 and fusogenic phenotypes in M-LECP to ensure both immuno-quiescence needed for regeneration and fusogenic capacity necessary for pro-mitotic reprogramming. 

## 5. Conclusions

**Figure 10:**
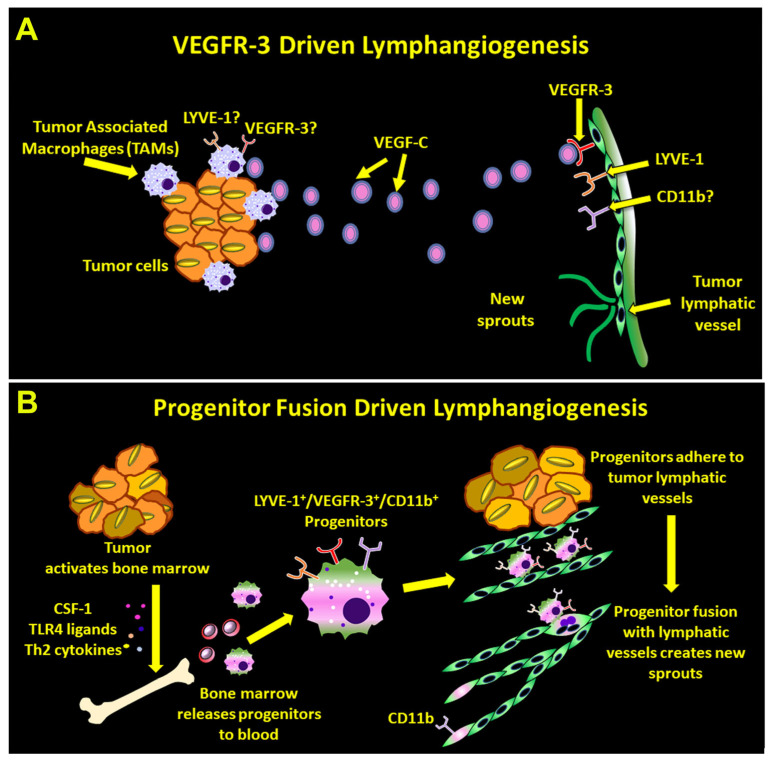
Mechanisms driving tumor lymphangiogenesis. The panels (**A**,**B**) illustrate the canonical and the new concept of tumor lymphatic formation, respectively. Note that the current understanding that primarily relies on VEGF-C/VEGFR-3 signaling (**A**) explains neither lymphatic marker expression by macrophages (e.g., LYVE-1) nor myeloid markers (e.g., CD11b) in new lymphatic vessels. In contrast, the new concept based on the evidence for progenitor fusion-driven lymphangiogenesis (**B**) explains both observations. This novel mechanism therefore expands the current view of tumor lymphatic formation.

In summary, we show that CSF-1, TLR4, and Th2 pathways collectively prepare myeloid–lymphatic progenitors to fuse with tumor LECs, leading to increased proliferation, lymphatic density, and LN metastasis. This is the first study demonstrating regulation of lymphatic vessel formation by progenitor-mediated fusion. While this mechanism departs from the current concept of exclusive reliance of lymphangiogenesis on the VEGFR-3 pathway, both mechanisms might be important for lymphatic outgrowth (Figure 10). Our data also show that pro-vascular and metastatic consequences of M-LECP fusion with LECs can be suppressed by exogenous drugs such as TLR4 inhibitors. This opens an avenue for new therapeutic approaches to control vascular formation based on understanding of the fusion process and its critical regulators. Since fusion is a common mechanism of progenitors for all lineages, this information can advance not only tumor and vascular biology but also tissue regeneration fields.

## Figures and Tables

**Table 1:** Expression of fusion markers in naïve and CSF-1-primed BM cells differentiated with LPS, IL-4, or IL-10.

Percent Positive Cells							
	Ex Vivo ^a^	LPS ^b^		IL-4 ^b^		IL-10 ^b^	
Marker	%	%	*p*-Value	%	*p*-Value	%	*p*-Value
Sirp-a	2.93 ± 0.05	80.69 ± 0.20	<0.0001	75.47 ± 0.62	<0.0001	71.80 ± 1.89	0.0008
Trem2	9.82 ± 2.06	85.19 ± 3.80	0.0020	62.43 ± 1.47	0.0023	41.76 ± 0.01	0.0041
Stab1	5.05 ± 0.64	48.40 ± 0.20	0.0001	59.36 ± 1.13	0.0003	75.20 ± 0.10	<0.0001
DC-Stamp	3.41 ± 0.08	26.88 ± 0.28	0.0001	51.05 ± 1.46	0.0009	11.94 ± 0.09	0.0002
CD36	4.60 ± 0.44	65.10 ± 0.20	<0.0001	65.08 ± 0.33	0.0001	10.78 ± 0.18	0.0057
CD47	18.29 ± 1.74	77.02 ± 0.48	0.0001	51.40 ± 2.46	0.0082	57.01 ± 2.67	0.0069
**Mean Fluorescent Intensity (MFI)**							
**Marker**	**MFI (×10^3^)**	**MFI (×10^3^)**	***p*-Value**	**MFI (×10^3^)**	***p*-Value**	**MFI (×10^3^)**	***p*-Value**
Sirp-a	31.62 ± 1.13	124.26 ± 2.66	0.0010	319.92 ± 8.00	0.0004	45.25 ± 0.58	0.0085
Trem2	21.72 ± 2.79	172.92 ± 15.32	0.0054	86.11 ± 1.25	0.0023	59.48 ± 1.58	0.0063
Stab1	14.62 ± 1.54	141.64 ± 0.87	0.0001	70.10 ± 1.36	0.0007	23.90 ± 0.60	0.0159
DC-Stamp	14.59 ± 3.75	79.39 ± 0.73	0.0034	16.95 ± 0.30	NS^c^	92.10 ± 11.03	0.0218
CD36	25.52 ± 4.21	79.55 ± 1.01	0.0064	79.55 ± 1.01	0.0064	78.53 ± 1.91	0.0057
CD47	41.17 ± 2.20	331.93 ± 37.63	0.0162	70.10 ± 1.36	0.0007	23.90 ± 0.60	0.0159

^a^ Freshly isolated bone marrow cells were analyzed by flow cytometry for indicated markers. ^b^ Bone marrow cells were primed with CSF-1 and treated with LPS or Th2 cytokines followed by flow cytometry analyses. ^c^ NS, not significant.

**Table 2:** Expression of fusogenic markers in M-LECPs recruited to MDA-MB-231 tumors in vivo.

Marker	Normal MFP	MDA-MB-231	*p*-Value	Reported Role in Fusion	Reference
CD36	26.89 ± 2.37 ^a^	74.10 ± 2.03	0.001	Binds to phosphatidylserine to facilitate cell adhesion	[57]
CD98	ND ^b^	86.03 ± 12.15		Promotes placental syncytia by binding to galectin-3	[70]
CD209	ND	86.99 ± 12.31		Promotes fusion of myeloid and virus-infected cells	[71]
Dap12	18.95 ± 3.05	86.58 ± 9.73	0.001	Mediates Dc-stamp and SIRP-a signaling that confers fusion competence of myeloid cells	[63]
DC-Stamp	25.95 ± 9.46	77.66 ± 4.71	0.001	Promotes multinucleation in IL-4-treated macrophages	[63]
Sirp alpha	28.92 ± 2.53	85.79 ± 16.21	0.001	Mediates Th2-dependent induction of key M2 and fusogenic traits	[61,74]
Sirp beta-1	ND	73.74 ± 10.94		Activates pro-fusogenic Dap12 signaling in myeloid cells	[73]
Stabilin-1	ND	98.92 ± 3.42		Binds to phosphatidylserine to facilitate cell adhesion	[65]

^a^ Percentage of Lyve-1+ cells expressing fusogenic markers of total M-LECPs recruited to normal MFPs or tumors. ^b^ ND, not determined.

## Data Availability

The data will be made available to interested parties upon request. Further queries can be directed to the corresponding author.

## Supplementary Materials

The following supporting information can be downloaded at: https://www.mdpi.com/article/10.3390/cancers17111804/s1, Supplementary Figure S1: Expression of fusogenic markers in M-LECPs recruited to human normal breast tissues; Supplementary Figure S2: Detection of BM-derived Y-chromosomes in CD206+ macrophages recruited to mouse normal MFPs. Supplementary Table S1: Antibodies used flow cytometry, immunohistochemistry, and neutralization; Supplementary Table S2: Human and mouse RT-qPCR primer sequences.

## Abbreviations

BC	Breast cancer
BM	Bone marrow
BMT	Bone marrow transplant
GFP	Green fluorescent protein
LEC	Lymphatic endothelial cells
LNs	Lymph nodes
LV	Lymphatic vessel
LVD	Lymphatic vessel density
MDSC	Myeloid-derived suppressor cell
M-LECP	Myeloid-lymphatic endothelial cell progenitor
TAMs	Tumor-associated macrophages
VEGFR-3	Vascular endothelial growth factor receptor-3
