# Supplementary material for: Bone Marrow Myeloid–Lymphatic Progenitors Expand Tumor Lymphatic Vasculature Through Cell Fusion
Source: Cancers (Basel). 2025 May 28;17(11):1804. doi: 10.3390/cancers17111804 (PMC12153582; doi:10.3390/cancers17111804)
Supplement: Supplementary file 1 [file cancers-17-01804-s001.zip › Supplemental Figures and Legends.pdf]

Supplemental Figure S1.

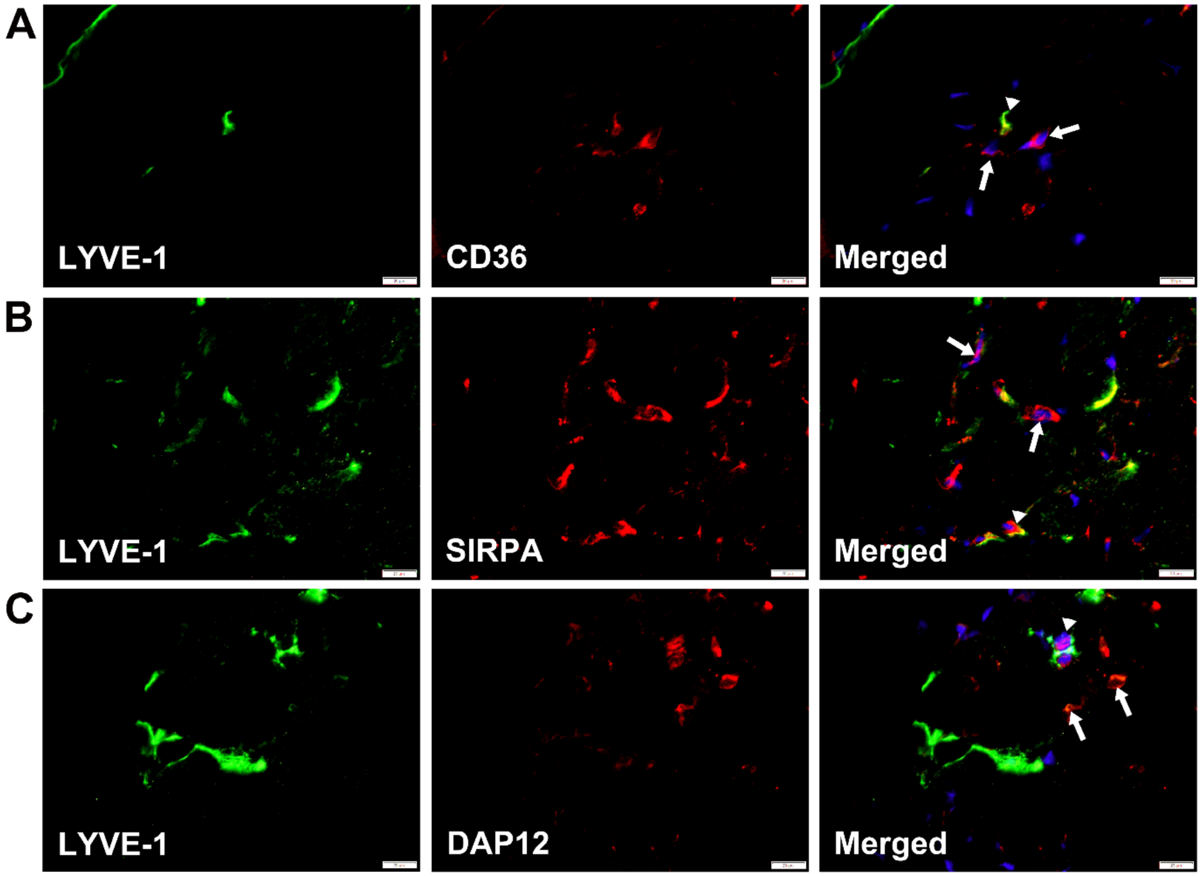

Supplemental Figure S2.

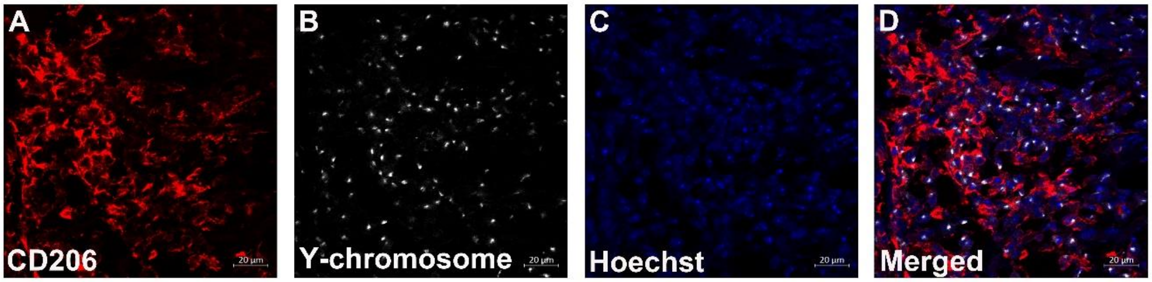

Supplemental Figure Legends

Supplemental Figure S1. Expression of fusogenic markers in M-LECP recruited to normal human breast tissues. Normal human breast specimens were co-stained with antibodies against LYVE-1 and fusogenic markers (A) SIRPA, (B) CD36 and (C) DAP12. White arrows point to cells that express fusogenic markers but not Lyve-1. White arrowheads point to cells that co-express LYVE-1 and fusogenic markers. Images were acquired at 400X magnification.

Supplemental Figure S2. Detection of BM-derived Y-chromosomes in CD206^{+} macrophages recruited to MDA-MB-231 tumors. Tumor sections were used for detection of Y-chromosomes and CD206 by FISH and immunostaining, respectively. Sections were counterstained with Hoechst dye to identify cells with nuclear Y-chromosome localization. White arrows point to double-positive cells within the tumor. Images were acquired at 400X magnification.
