# Supplementary material for: Bone Marrow Myeloid–Lymphatic Progenitors Expand Tumor Lymphatic Vasculature Through Cell Fusion
Source: Cancers (Basel). 2025 May 28;17(11):1804. doi: 10.3390/cancers17111804 (PMC12153582; doi:10.3390/cancers17111804)
Supplement: Supplementary file 1 [file cancers-17-01804-s001.zip › Table S1_Antibodies_Dec 8, 2024.pdf]

Table S1 Antibodies used for flow cytometry, immunohistochemistry and neutralization

Antigen	Description	Vendor	Catalog no.	Concentration µg/ml IF	Flow cytometry	NT
Acetyl-Histone 3	Rabbit anti-human/ mouse	Upstate	06-599	1	N/A	N/A
CD36	Rabbit anti-human/mouse	Caymen Chemical	100011	0.5	5	N/A
CD47	Rat anti-mouse CD47; clone miap301	BioLegend	127502	N/A	5	N/A
CD206	Goat anti-mouse	Thermo Fisher	PA546994	0.2	N/A	N/A
DAP12	Rabbit anti-human/mouse	Millipore Sigma	HPA041899	0.3	N/A	N/A
DC-Stamp	Rabbit anti-human/mouse	My BioSource	MBS7109052	1	5	N/A
FITC	Mouse anti-FITC	Jackson ImmunoResearch	200-002-037	1	N/A	N/A
IL-4	Rat anti-mouse; clone 11B11	BioXCell	BE0045	N/A	N/A	5
IL-10	Rat anti-mouse; clone JES5-2A5	BioXCell	BE0049	N/A	N/A	5
LYVE-1	Goat anti-human	R&D Systems	AF2089	0.5	N/A	N/A
Lyve-1	Goat anti-mouse	R&D Systems	AF2125	0.5	N/A	N/A
Meca-32	Rat anti-mouse, clone MECA-32	BioXCell	BE0200	0.5	N/A	N/A
SIRPA	Rabbit anti-human	Millipore Sigma	HPA058511	0.05	N/A	N/A
Sirpa	Rat anti-mouse; clone PG4	BioXCell	BE0322	1	5	N/A

Stab1	Sheep anti-human/mouse	R&D Systems	AF3825	1.5	5	N/A
Trem2	Rabbit anti-human/mouse	Bioss	bs-2723R	N/A	5	N/A

DC-Stamp, dendritic cell-specific transmembrane protein; IF, immunofluorescence; LYVE-1, lymphatic vessel endothelial hyaluronan receptor 1; NT, neutralization; SIRPA, signal regulatory protein alpha; Stab1, stabilin-1; Trem2, triggering receptor expressed on myeloid cells 2.
