# Supplementary material for: Bone Marrow Myeloid–Lymphatic Progenitors Expand Tumor Lymphatic Vasculature Through Cell Fusion
Source: Cancers (Basel). 2025 May 28;17(11):1804. doi: 10.3390/cancers17111804 (PMC12153582; doi:10.3390/cancers17111804)
Supplement: Supplementary file 1 [file cancers-17-01804-s001.zip › Table S2-primers Dec 9,2024.pdf]

Table S2   Human and mouse RT-qPCR primer sequences

Human

Gene[a]	Size (bp)	Forward sequence	Reverse sequence
ACTB	131	5'-TCCTCTCCCAAGTCCACACAGG-3'	5'-GGGCACGAAGGCTCATCATTC-3'
ANO6	113	5'-AATGGAGGAGGAGGAGGACG-3'	5'-CCGGGGTTCGAAAATCATGC-3'
CD36	92	5'-GGCTGTGACCGGAACTGTG-3'	5'-AGGTCTCCAACTGGCATTAGAA-3'
CD47	164	5'-GCGGCGTGTATACCAATGC-3'	5'-TTCAGTTATTCATTAAGGGGTTCCT-3'
CD63	75	5'-ATGCAGGCAGATTTTAAGTGCT-3'	5'-GTTCTTCGACATGGAAGGGATTT-3'
CD163	89	5'-GCGGGAGAGTGGAAGTGAAAG-3'	5'-GTTACAAATCACAGAGACCGCT-3'
CD204	151	5'-ACCCCCGGGTGAAAAA-3'	5'-TGGCCTTCCGGCATA-3'
CD206	105	5'-CTACAAGGGATCGGGTTTATGGA-3'	5'-TTGGCATTGCCTAGTAGCGTA-3'
CD280	99	5'-CCGAAACCGGCTATTCAACCT-3'	5'-CGGTCACACTCATACATGCCC-3'
CLEC5A	208	5'-AGCATTAGGCCACCAGGAAG-3'	5'-CCTGGTGGTGGTGAAACCAT-3'
DAP12	228	5'-TGGCTGTAAGTGATTGCAGTTG-3'	5'-CGCTGTAGACATCCGACCTC-3'
DC-SIGN	106	5'-GAAGGCACGTGGCAATG-3'	5'-ATTCCGCGCAGTCTTCC-3'
DC-SIGNR[b]	273	5'-ACTTCATGTCTAACTCCCAGCG-3'	5'-ATTCCGCACAGTCTTCATTCC-3'
DC-STAMP	157	5'-CCTTGCCACTCCACTAAGTGT-3'	5'-CTCTGTGGTTGTTGCCATCTG-3'
MGL1[c]	195	5'-TGGGAATCACACCCTCCAGA-3'	5'-AGGAGGGACTGGAGAGGAAG-3'
SIRPA	211	5'-AAATACCGCCGCTGAGAACA-3'	5'-TGTGATATCATTTGTGTCCTGTGT-3'
STAB1	244	5'-CAGAGGGTAGACGTGATGGC-3'	5'-TTAGCACATTGAGCCCCGTT-3'
TREM2	127	5'-TCTCCAGGGCTGAGAGACAC-3'	5'-TGCCAGAGCAGAACAAGGAG-3'

Mouse

Gene[a]	Size (bp)	Forward sequence	Reverse Sequence
Actb	153	5'-GGCTGTATTCCCCTCCATCG-3'	5'-CCAGTTGGTAACAATGCCATGT-3'
Ano6	150	5'-AGGATGGAGACATTGGTGATGT-3'	5'-TCCTGAAGTCCTGCTGATTCT-3'
Cd36	91	5'-GCGACATGATTAATGGCACA-3'	5'-CCTGCAAATGTCAGAGGAAA-3'
Cd47	210	5'-GGAGCCATCCTTCTCATCCC-3'	5'-TGCCATGATGCAGAGACACA-3'
Cd63	138	5'-AGAGACCAGGTGAAGTCAGAG-3'	5'-AGTCTGTGTAGTTAGAAGCTCCA-3'
Cd163	108	5'-GGTGGACACAGAATGGTTCTTC-3'	5'-CCAGGAGCGTTAGTGACAGC-3'
Cd204	166	5'-AGTGCTGTCTTCTTTACCAGCA-3'	5'-CTGAAGGGAGGGGCCATTTT-3'
Cd206	88	5'-GAGGGAAGCGAGAGATTATGGA-3'	5'-GCCTGATGCCAGGGTAAAGCA-3'
Cd280	98	5'-GCTTCTGCCCCATCAAGAGTA-3'	5'-AGGACAGTGTGGATTGGAAGT-3'
Clec5a	259	5'-GGACATTACCGAGCAGGAGC-3'	5'-TGGGGACGAAGCCATCATTAC-3'
Dap12	291	5'-GATTGCCCTGGCTGTGTACT-3'	5'-GGGAGGTACCCTGTGGATCT-3'
Dc-sign	110	5'-GTTTGTTCTGTGCTGCTGGTT-3'	5'-GCCTTCAACTGGGTCAGTTCT-3'
Dc-signr[b]	287	5'-CTGACAGATGAGCTTACGTCCA-3'	5'-CACAGGCGGAAGAGTTCAGTC-3'
Dc-stamp	188	5'-CTGTTGCTTTGTGGCCTTCC-3'	5'-AAGCGTTCCTACCTTCACGG-3'
Mgl2	211	5'-GACTGAGTTCTCGCCTCTGG-3'	5'-CTGGGAAGGAATTAGAGCAAACT-3'
Sirpa	121	5'-CTCGTAGTCCTGCTGATGGC-3'	5'-TCTGGGTTATTTCCCTGGCG-3'
Stab1	146	5'-GGCAGACGGTACGGTCTAAAC-3'	5'-AGCGGCAGTCCAGAAGTATCT-3'
Trem2	286	5'-CGGAATGGGAGCACAGTCAT-3'	5'-GGTAGGCTAGAGGTGACCCA-3'

[a] Primers were designed based on human or mouse CDS of targets found in NCBI database. All primers were validated using human or mouse universal cDNA, respectively. Primers were confirmed to exclusively detect specie-specific cDNA.

[b] Alias CLEC4M, C-type lectin domain family 4, member M.

[c] Alias CLEC10A, C-type lectin domain family 10, member A.

Ano6, anoctamin 6; Clec5a, C-type lectin domain family 5, member A; DC-SIGN, dendritic cell-specific ICAM-3-grabbing non-integrin 1; DC-SIGNR, dendritic cell-specific ICAM-3-grabbing non-integrin 1-related protein; Mgl2, macrophage galactose-type C-type lectin 2.
